# Long-Term Outcomes and Rebleeding Risk Factors in Suspected Small Bowel Bleeding: A Study Based on the Modified Saurin Classification

**DOI:** 10.1007/s10620-025-09640-5

**Published:** 2026-01-04

**Authors:** Sachiyo Onishi, Kiichi Otani, Naoya Masuda, Hiroki Taniguchi, Kentaro Kojima, Jun Takada, Masaya Kubota, Takashi Ibuka, Masahito Shimizu

**Affiliations:** Gifu University School of Medicine Graduate School of Medicine, Endoscopy, 1-1 Yanagido, Gifu City, 501-1193 Japan

**Keywords:** Obscure gastrointestinal bleeding (OGIB), Suspected small bowel bleeding (SSBB), Modified Saurin classification, Balloon-assisted endoscopy, Capsule endoscopy

## Abstract

**Background:**

Small-bowel endoscopy is essential for diagnosing and managing suspected small bowel bleeding (SSBB); however, rebleeding after treatment remains clinically challenging. Although several rebleeding prediction models have been proposed, few are based on endoscopic findings, and data on long-term outcomes are limited.

**Aims:**

This study aimed to evaluate rebleeding risk factors and long-term outcomes using the modified Saurin classification (modified SC), which is compatible with balloon-assisted endoscopy (BAE).

**Methods:**

We retrospectively evaluated 278 consecutive patients with OGIB who underwent capsule endoscopy (CE) or BAE between October 2008 and December 2023. The lesions were categorized using modified SC as P0 (*n* = 58), P1 (*n* = 109), P2 (*n* = 71), or no findings (*n* = 40). Treatment was classified as endoscopic or definitive (surgical or interventional radiology).

**Results:**

Of the 278 patients (mean age 67.7 years; 150 males), 158 had occult OGIB and 120 had overt OGIB. Treatment was administered to 73 patients (46 endoscopic and 27 definitive cases). Over a mean follow-up of 2.2 years, 32 patients (11.5%) experienced rebleeding. Multivariate analysis identified modified SC P1 lesions (odds ratios [ORs], 2.33; *p* = 0.03), antiplatelet drugs use (ORs 3.02, *p* = 0.04) and treatment interventions (ORs, 2.28; *p* = 0.02) as independent predictors. The 3-year rebleeding rate was higher in P1 (27.2%) than in P0 (9.5%) and P2 (13.8%). Rebleeding was more frequent in the endoscopic group (39.9%) than in the definitive group (10.5%; *p* = 0.04).

**Conclusion:**

Modified SC P1 lesions were significantly associated with rebleeding. Proactive treatment strategies and long-term follow-up are crucial for effective management of SSBB.

**Supplementary Information:**

The online version contains supplementary material available at 10.1007/s10620-025-09640-5.

## Introduction

Obscure gastrointestinal bleeding (OGIB) is a relatively uncommon clinical condition, accounting for approximately 5% of all gastrointestinal bleeding cases [1]. OGIB can arise from any segment of the gastrointestinal tract; however, when the bleeding source is small, intermittent, or difficult to visualize, the diagnosis becomes particularly challenging.

The introduction of small bowel capsule endoscopy (SBCE) and balloon-assisted endoscopy (BAE) has significantly improved the diagnostic yield for small bowel bleeding. SBCE is a noninvasive modality that allows complete visualization of the small intestine and is widely used as an initial diagnostic tool in patients with OGIB [2, 3]. In contrast, BAE offers the advantage of enabling direct observation and therapeutic interventions such as endoscopic hemostasis. Despite technological advances [4], patients with OGIB often undergo multiple diagnostic procedures, require prolonged hospitalization, and more frequent blood transfusions than those with upper or lower gastrointestinal bleeding. This contributes to considerable consumption of healthcare resources. Although OGIB has been the subject of extensive research over the past several decades, significant challenges remain in both its diagnostic approach and management.

Once the bleeding source is identified and treated appropriately, the short-term prognosis is generally favorable [5]. However, failure to identify the source of bleeding is associated with a high risk of bleeding. This concern is particularly pronounced in cases of overt OGIB, in which spontaneous hemostasis may be achieved initially, but recurrent bleeding can potentially be fatal [1, 6–8].

In 2003, Saurin et al. [9] proposed a classification system that stratified lesions observed on CE into three categories based on their bleeding potential. The Saurin classification has since been widely adopted, and several international guidelines have been recommended, including those of the European Society of Gastrointestinal Endoscopy (ESGE) [4], to guide post-SBCE management strategies. However, despite the frequent use of BAE for both diagnostic and therapeutic purposes, a standardized system for risk stratification based on BAE findings has yet to be established.

In light of these considerations, we devised a novel index, the modified Saurin classification (modified SC), based on the original Saurin classification, which can be applied to the BAE findings. The aim of the present study was to evaluate the association between the modified SC and long-term outcomes in patients with suspected small bowel bleeding (SSBB) and to clarify the clinical utility of this new classification system.

## Methods

### Study Design and Patient Selection

The retrospective observational study was conducted at Gifu University Hospital, in compliance with the ethical standards outlined in the Declaration of Helsinki, and was approved by the Institutional Review Board of Gifu University Hospital.

This study included 278 patients diagnosed with SSBB between October 2008 and December 2023. SSBB was defined as gastrointestinal bleeding of unknown origin that persisted even after negative esophagogastroduodenoscopy (EGD) and colonoscopy (CS).

Eligible patients underwent SBCE and/or BAE during the diagnostic workup for SSBB. Patients with incomplete endoscopic data or insufficient clinical information were also excluded.

### Endoscopic Evaluation

SBCE was performed using a PillCamTM SB3 capsule (Covidien, Mansfield, MA, USA). In cases where SBCE preceded BAE, the route of insertion (oral or anal) was determined based on the location of the suspected lesion identified by SBCE. For patients who did not undergo SBCE, the approach was determined based on contrast-enhanced computed tomography (CT).

When the source of bleeding could not be identified during the initial procedure, a second-look BAE was performed via the alternative route on a separate day to achieve total enteroscopy. For retrograde BAE, bowel preparation with standard oral purgatives was performed before the procedure.

When both SBCE and BAE were performed in the same patient, CE findings were primarily used for initial detection and localization of lesions, whereas BAE served as the definitive modality for lesion characterization and final classification.

### Modified Saurin Classification

**Fig. 1:**
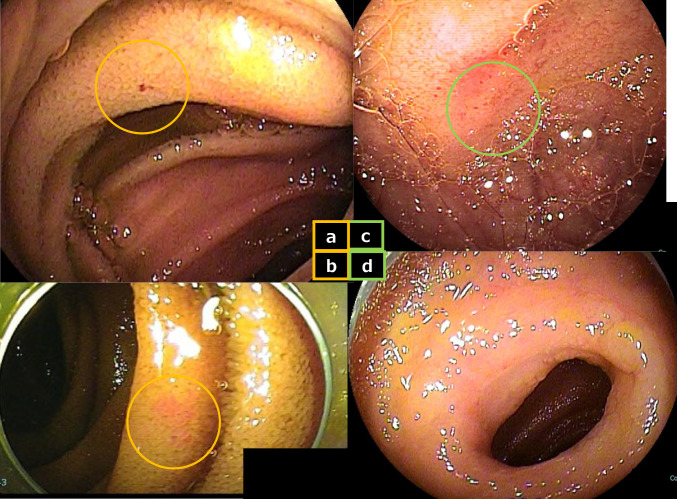
Findings of modified Saurin Classification (modified SC) 0 are shown. These include red spots (**a**), erythematous patches (**b**), villous loss (**c**), and scars (**d**). Red spots (a) and erythematous patches (b) were classified as vascular lesions, whereas villous loss (c) and scars (d) were classified as mucosal lesions

To evaluate the clinical relevance of the lesions detected using BAE, we created a modified version of the Saurin classification (modified SC) originally proposed by Saurin et al. in 2003 for SBCE, adapted for use with CE and BAE. Although the original Saurin Classification classifies lesions into three categories–P0, P1, and P2–based on their presumed bleeding potential, no such standardized system has been established for BAE findings. Therefore, we adapted the original criteria for use with BAE as follows:

P0 (Fig. 1): lesions considered to have a low risk of clinical bleeding, including red spots, mild mucosal erythema (vascular lesions), and villous atrophy or mucosal scars (mucosal lesions).

**Fig. 2:**
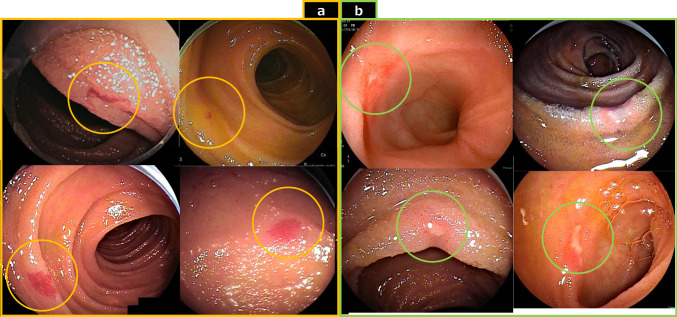
Findings of modified SC P1 are shown, including ectasia (**a**) and small erosion (**b**). Ectasia (a) was classified as a vascular lesion, and a small erosion (b) as a mucosal lesion

P1 (Fig. 2): Lesions with uncertain but possible risk of bleeding, including ectasia or small mucosal erosion. The definitions of ectasia [10] and small erosion [11] in this study were adopted from previously published.

**Fig. 3:**
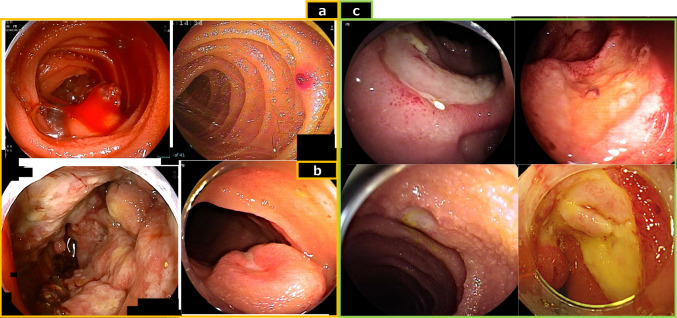
Findings of modified SC P2 are shown. These include angiomas (**a**), neoplastic lesions (**b**), and mucosal ulcers (**c**). Angiomas were classified as vascular lesions, and mucosal ulcers as mucosal lesions

P2 (Fig. 3): Lesions with a high likelihood of clinical bleeding that typically require therapeutic intervention, including angiomas, mucosal ulcers, and neoplastic lesions. The definitions of mucosal ulcers in this study were adopted from adopted from previously published studies [11].

No findings: No findings satisfying P0 to P2 were observed.

In this study, therapeutic intervention comprised endoscopic therapy—mainly clipping at our institution—and definitive therapy, which included interventional radiology–guided embolization and surgery.

All CE and BAE examinations were interpreted using a standardized institutional double-check system. The primary endoscopist generated an initial report, after which a second board-certified endoscopist within the gastroenterology team reviewed the findings. Any areas of uncertainty, including lesion type, vascular features, and assignment of the modified SC (P0/P1/P2), were discussed among the reviewing endoscopists until a consensus interpretation was reached.

### Outcomes and Follow-Up

The primary outcome was the association between modified SC and risk of bleeding. Secondary outcomes included associations between the modified SC and other clinical factors, such as the presence of liver cirrhosis, need for endoscopic therapy, and long-term prognosis.

Rebleeding was defined as the occurrence of a clinically evident bleeding episode, a decrease in hemoglobin of ≥ 2 g/dL, and the need for repeat endoscopy, as adjudicated by a team of gastroenterologists based on clinical history and laboratory findings. Some events required hospitalization or blood transfusion, although these were not mandatory criteria. No case required surgical intervention for rebleeding during the study period. This standardized adjudication process ensured consistency and reproducibility in the assessment of rebleeding events.

All patients were followed until the occurrence of rebleeding or censoring. Censoring date: April 2, 2025. The median follow-up duration was 0.97 years (IQR 0.00–14.50). Follow-up data were obtained through outpatient visits and review of hospital records.

### Statistical Analysis

Categorical variables were presented as frequencies and percentages, and continuous variables as means ± standard deviations or medians with interquartile ranges, as appropriate. The primary analysis was performed using multivariable logistic regression to identify predictors of the occurrence of rebleeding during available follow-up. Covariates were selected a priori on clinical grounds and included cirrhosis, antiplatelet drugs use, hemoglobin (10.5 g/dl), modified SC (P1 vs other), therapeutic intervention (endoscopic vs non-endoscopic), age, number of lesion (multiple or not), and bleeding type(overt/occult). Kaplan–Meier curves with log-rank tests are presented to descriptively illustrate cumulative incidence of rebleeding over time across exposure groups; these were not used to derive the primary estimates of association. Statistical significance was set at *p* < 0.05. All statistical analyses were performed using JMP software (SAS Institute Inc., Cary, NC, USA).

## Results

### Patient Characteristics

The mean age of patients included in the study was 67.7 years, and 54.0% were men. The mean BMI was 22.2 kg/m^2^. At diagnosis, the mean hemoglobin level was 9.2 g/dL, albumin 3.6 g/dL, serum iron 74.0 μg/dL, and C-reactive protein (CRP) 0.64 mg/dL. Overt bleeding type was observed in 56.8% of patients and occult bleeding type in 43.2% (Table 1).

**Table 1:** Baseline characteristics

Characteristic	
Mean age, (± SD), years	67.7 (± 14.2)
Sex, *n* (%)	
Male	150 (54.0)
Female	128 (46.0)
BMI, (± SD), kg/m^2^	22.2 (5.5)
Hemoglobin level, (± SD), g/dL	9.2 (± 2.5)
Albumin, (± SD), g/dL	3.6 (± 0.7)
Serum iron, (± SD), μg/dL	74.0 (± 75.9)
CRP, (± SD), mg/dL	0.64 (± 1.51)
Bleeding type, *n* (%)	
Overt	158 (56.8)
Occult	120 (43.2)
Method	
CE	110 (39.5)
BAE	83 (30.0)
In combination	85 (30.5)
Medication	
Antiplatelets	65 (23.3)
Anticoagulants	50 (18.0)
NSAIDs	87 (31.3)
Underlying disease, *n* (%)	
Cerebrovascular disease	21 (7.6)
Liver cirrhosis	32 (23.3)
Dialysis	15 (18.0)
Myocardial infarction	51 (18.4)
Chronic atrial fibrillation	27 (9.7)
Malignant tumor	70 (25.2)
Diabetes mellitus	44 (15.8)

*BMI* body mass index, *CRP* C-reactive protein, *CE* capsule endoscopy, *BAE* balloon-assisted endoscopy, *NSAIDs* nonsteroidal anti-inflammatory drugs

SBCE alone, BAE alone, and both procedures were performed in 39.5%, 30.0%, and 30.5% of cases, respectively. NSAIDs, antiplatelets, and anticoagulants were used in 31.3%, 23.3%, and 18.0% of patients, respectively. Major comorbidities included malignancy (25.2%), cerebrovascular disease (7.6%), and liver cirrhosis (11.5%). Modified SC yielded P0 (20.8%), P1 (39.2%), P2 (25.5%), or no findings (14.4%). The main lesion types were mucosal (37.4%), vascular (34.5%), tumor (9.4%), and diverticular (4.3%) (Table 1).

Specific treatments were administered to 26.0% of all cases. Endoscopic hemostasis was performed in 46 patients, and definitive treatment was performed in 27 patients (Table 2). Specific treatment was performed in 5 patients (5.2%) with P0 lesions (4 endoscopic, 1 definitive), in 32 patients (29.4%) with P1 lesions (30 endoscopic, 2 definitive), and in 36 patients (50.7%) with P2 lesions (12 endoscopic, 24 definitive).

**Table 2:** Background of baseline lesions

Variables	*n* (%)
Modified SC	
P0	58 (20.8)
P1	109 (39.2)
P2	71 (14.4)
No findings	40 (14.4)
Identified Lesion	
Vascular lesion	96 (34.5)
Mucosal lesion	104 (37.4)
Tumor	26 (9.4)
Diverticula	12 (4.3)
Therapeutic intervention	73 (26.2)

*SC* Saurin classification

### Overall and Modified Saurin Classification-Specific Rebleeding Rate

Rebleeding was observed in 32 patients (11.5%) during the follow-up period. When stratified by modified SC, the rebleeding rates were as follows: 5.1% in the P0 group, 19.3% in the P1 group, 7.0% in the P2 group, and 7.5% in patients with no identifiable small bowel findings. Notably, the P1 group exhibited the highest rebleeding rate among all patient groups.

### Comparison of Background Factors in Patients with Rebleeding

To evaluate potential predictors of rebleeding, patients who experienced rebleeding and those who did not were compared. No statistically significant differences between the two groups were observed in terms of age, sex, or underlying comorbidities, including cardiovascular disease, liver cirrhosis, diabetes mellitus, or atrial fibrillation. Additionally, hematological parameters, including hemoglobin levels and other laboratory values, were not significantly associated with the risk of rebleeding. The use of NSAIDs, antiplatelet agents, anticoagulants, proton pump inhibitors (PPIs), and other acid-suppressing agents was not significantly associated with rebleeding. However, a notable finding was that patients classified as having modified SC P1 had a significantly higher rate of rebleeding, which is important for identifying lesions that could cause rebleeding. Furthermore, patients who underwent endoscopic treatment during the endoscopic evaluation exhibited a significantly increased risk of rebleeding (Table 3).

**Table 3:** Comparison of baseline characteristics according to rebleeding status

*N*	Rebleeding	No rebleeding	*p* value
	*n* = 32	*n* = 246	
Age (± SD), years	70.4 ± 12.0	68.3 ± 13.0	0.41
Sex (male/female), *n*	13/19	137/109	0.13
Cerebrovascular disease, *n* (%)	3 (9.4)	18 (7.3)	0.72
Liver cirrhosis, *n* (%)	5 (15.6)	27 (10.9)	0.38
Dialysis, *n* (%)	1 (3.13)	14 (5.69)	1.00
Chronic atrial fibrillation, *n* (%)	4 (12.5)	23 (9.4)	0.53
Diabetes mellitus, *n* (%)	6 (18.8)	38 (15.5)	0.61
Alb (± SD), g/dL	3.6 ± 0.71	3.62 ± 0.69	0.76
Hb (± SD), g/dL	8.5 ± 2.6	9.3 ± 2.4	0.06
CRP, mg/dL	0.42 ± 0.77	0.78 ± 1.16	0.36
NSAIDs, *n* (%)	11 (34.4)	76 (30.9)	0.69
Antiplatelets, *n* (%)	5 (15.6)	54 (24.4)	0.48
Anticoagulants, *n* (%)	4 (12.5)	46 (18.7)	0.47
PPI/H2RA, *n* (%)	19 (59.4)	132 (53.6)	1.00
Vascular lesion, *n* (%)	15 (46.9)	80 (32.5)	0.09
Modified SC P1, *n* (%)	21 (65.6)	88 (35.7)	< 0.01
Therapeutic intervention, *n* (%)	16 (50.0)	57 (23.1)	< 0.01
Multiple lesions, *n* (%)	16 (50.0)	90 (36.6)	0.17

*Alb* albumin, *Hb* hemoglobin, *CRP* C-reactive protein, *NSAIDs* non-steroidal anti-inflammatory drugs, *PPI* proton pump inhibitors, *H2RA* H2 receptor antagonist, *SC* Saurin classification

### Cumulative Rebleeding Rate

**Fig. 4:**
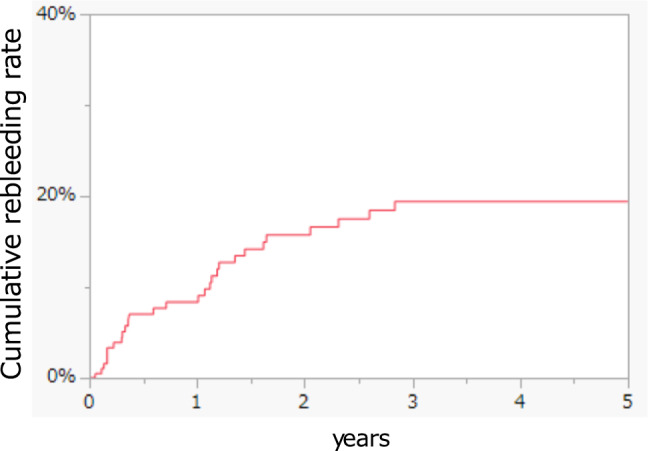
Cumulative rebleeding rates for all patients are shown. The cumulative rebleeding rate was 9.1% at 1 year, 15.7% at 2 years, and 19.4% at 3 years following the initial evaluation

During the follow-up period, the cumulative incidence of rebleeding gradually increased. Specifically, the cumulative rebleeding rates were 9.1% at 1 year, 15.7% at 2 years, and 19.4% at 3 years after the initial evaluation. This time-dependent trend highlights the importance of continued clinical monitoring, even in patients who remain stable during the early postendoscopic period (Fig. 4).

### Predictive Factors for Rebleeding

Multivariate logistic regression analysis was performed using liver cirrhosis, antiplatelet use, baseline hemoglobin levels, modified SC, presence of endoscopic treatment, age, multiple lesion, and bleeding type as covariates. The analysis identified antiplatelet drugs use (odds ratios [ORs] 3.02(1.01–9.05) *p* = 0.04), modified SC P1 (ORs 2.49(1.00–6.28), *p* = 0.04) and endoscopic treatment (ORs 4.56(1.88–11.2), *p* =  < 0.01) as independent predictors of rebleeding (Table 4).

**Table 4:** Predictive factors for rebleeding; multivariate analysis

Variable	ORs (95%CI)	*p* value
Liver cirrhosis	1.4 (0.43–4.50)	0.57
Antiplatelet drugs use	3.02 (1.01–9.05)	0.04
Hemoglobin level (< 10.5 g/dl)	1.95 (0.67–5.66)	0.34
Modified SC of P1	2.49 (0.99–6.28)	0.04
Bleeding type, overt	1.12 (0.49–2.55)	0.78
Age (> 75 years)	1.52 (0.67–3.45)	0.32
Multiple lesion	1.20 (0.50–2.88)	0.67
Endoscopic treatment	4.56 (1.88–11.2)	< 0.01

*ORs* odds ratios, *CI* confidence interval, *SC* Saurin classification

In the sensitivity analysis restricted to BAE-only patients, modified SC remained the only independent predictor of rebleeding. Full results are provided in Supplementary Table 1.

Furthermore, an Inverse Probability of Treatment Weighting (IPTW) -adjusted sensitivity analysis was conducted. After weighting, cirrhosis, age, and antiplatelet therapy emerged as significant predictors, while the modified SC remained independently associated with rebleeding (OR 2.60, 95% CI 1.59–12.4). (Table 5).

**Table 5:** Inverse Probability of Treatment Weighting (IPTW) -adjusted sensitivity analysis

Variable	ORs (95%CI)	*p* value
Liver cirrhosis	2.88 (1.67–4.99)	< 0.01
Antiplatelet drugs use	2.82 (1.53–5.21)	< 0.01
Hemoglobin level (< 10.5 g/dl)	1.11 (0.67–1.80)	0.69
Modified SC of P1	2.60 (1.59–12.4)	< 0.01
Bleeding type, overt	1.37 (0.67–1.80)	0.68
Age (> 75 years)	2.31 (1.50–3.49)	< 0.01
Multiple lesion	1.33 (0.81–2.17)	0.24

*ORs* odds ratios, *CI* confidence interval, *SC* Saurin classification

**Fig. 5:**
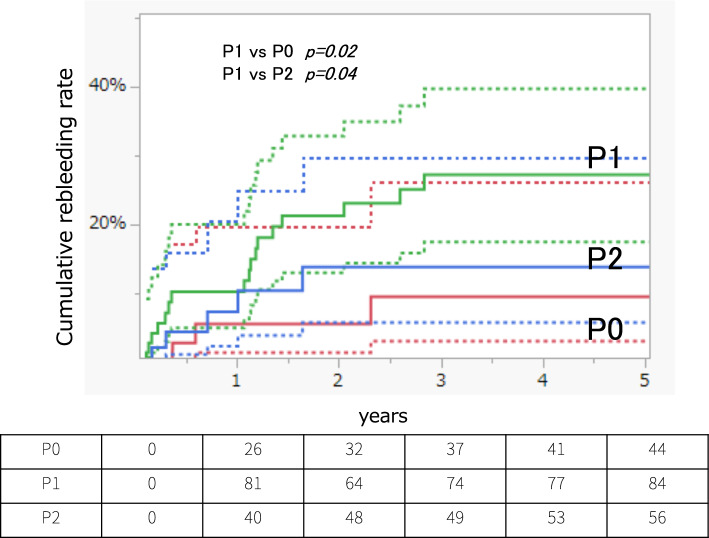
Cumulative rebleeding rate by modified SC. Modified SC P1 had a significantly higher bleeding rate than P0 and P2

When stratified by the modified SC, patients classified as P1 exhibited significantly higher cumulative rebleeding rates than those in the other groups. (P1 vs P0; *p* = 0.02, P1 vs P2; *p* = 0.04). Specifically, the cumulative rebleeding rates in the P1 group were 11.8% at 1 year, 21.3% at 2 years, and 27.2% at 3 years, indicating a sustained risk of rebleeding in this subgroup (Fig. 5).

**Fig. 6:**
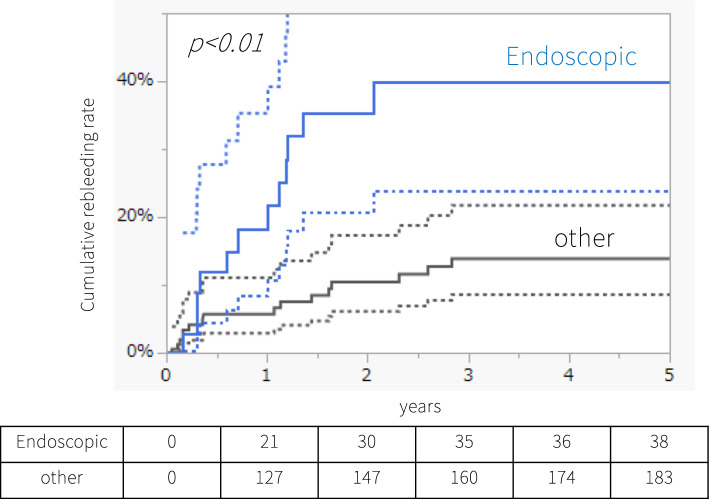
Cumulative rebleeding rate stratified by treatment. The cumulative rebleeding rate was significantly higher after endoscopic treatment than after other treatments

Similarly, an analysis based on therapeutic interventions revealed that patients who underwent endoscopic hemostasis experienced significantly higher cumulative rebleeding rates than those who did not (*p* < 0.01). In this group, the cumulative rebleeding rates were 18.3% at 1 year, 35.3% at 2 years, and 39.9% at 3 years (Fig. 6).

Further comparison of the baseline characteristics between patients with and without therapeutic intervention showed that the intervention group had a significantly higher prevalence of antiplatelet agent use. In addition, multiple vascular lesions were significantly more common in this group, suggesting that patients who underwent endoscopic hemostasis tended to have more complex or higher-risk sources of bleeding (Table 6).

**Table 6:** Comparison of baseline characteristics between patients with and without therapeutic intervention

Characteristic	Endoscopic	Other	*p* value
	46	(Definitive, *n* = 27; Follow, *n* = 205)	
Antiplatelet drugs, *n* (%)	17 (36.9)	48 (20.7)	0.02
Anticoagulants, *n* (%)	13 (28.3)	37 (15.9)	0.05
NSAIDs, *n* (%)	20 (43.5)	67 (28.9)	0.05
Cerebrovascular disease, *n* (%)	4 (8.7)	17 (7.3)	0.76
Liver cirrhosis, *n* (%)	4 (8.7)	28 (12.1)	0.62
Myocardial infarction, *n* (%)	12 (26.1)	39 (16.8)	0.15
Chronic atrial fibrillation, *n* (%)	6 (13.0)	21 (9.1)	0.41
Multiple lesions, *n* (%)	23 (50.0)	78 (33.6)	0.04
Vascular lesion, *n* (%)	30 (65.2)	66 (28.5)	< 0.01
Mucosal lesion, *n* (%)	12 (26.1)	92 (39.6)	0.09

*NSAIDs* non-steroidal anti-inflammatory drugs

## Discussion

In this study, we investigated the stratification of rebleeding risk based on the modified SC by analyzing the clinical characteristics, endoscopic findings, and long-term outcomes of patients with SSBB. Our results demonstrated that lesions classified as P1 by the modified SC, which have traditionally been considered to have a low or uncertain risk of active bleeding, were significantly associated with the risk of rebleeding. These findings have significant clinical implications. Our results are consistent with those previously reported by Hirata et al. [12], who identified P1 lesions and endoscopic hemostasis as independent predictors of rebleeding based on SBCE findings. In line with these findings, we demonstrated that P1 lesions identified not only by SBCE but also by BAE were predictive of rebleeding.

In daily clinical practice, the risk of bleeding is often driven more by the endoscopic characteristics of a lesion—such as morphology, vascularity, and depth—than by its pathological classification. For instance, vascular lesions, superficial erosions, and small neoplastic lesions arise from different etiologies, yet they may exhibit comparable bleeding potential when their endoscopic appearance is taken into account. From this perspective, categorizing lesions based on their likelihood of causing bleeding rather than on disease type offers a more practical and consistent approach to risk stratification. Therefore, we believe that the modified SC reflects these clinically relevant differences and provides a meaningful framework for assessing rebleeding risk in SSBB.

In our study, we defined “no abnormal findings” as the absence of P0–P2 lesions, based on the understanding from a report by Hirata et al. [12] that even minor abnormalities may carry a risk of bleeding. In previous studies, the definition of “no abnormal findings” varied, with some studies classifying P0 only or both P0 and P1 under this category. Our study is unique in that it evaluated rebleeding rates by clearly distinguishing between truly negative SBCE findings and SBCE-positive findings, a distinction not commonly made in earlier reports. Furthermore, few studies have examined the long-term outcomes of OGIB according to the Saurin classification findings. In a previous report by Cho et al. [13], the cumulative rebleeding rates for P0 lesions at 1, 2, and 5 years were 9.2, 25.4, and 25.4%, respectively, whereas those for P1 lesions were 6.9, 11.8, and 18.6%, with no significant differences between P0 and P1. Conversely, in a study by Hirata et al., the cumulative rebleeding rates for P0 lesions at two and five years were 1 and 3%, respectively, whereas those for P1 lesions were 4 and 11%, with P1 showing a significantly higher rebleeding rate. The authors also reported that P1 lesions were independent predictors of rebleeding based on SBCE findings [12].

Taken together, our findings further support the predictive value of P1 lesions for rebleeding based on endoscopic findings assessed by modified SC and not limited to SBCE alone. In other words, even minor findings may be associated with the risk of rebleeding in patients with SSBB.

Several previous studies have investigated predictive factors for rebleeding in patients with OGIB. The reported risk factors include female sex [14], dialysis [15], liver cirrhosis with portal hypertension [14, 15], warfarin use (anticoagulants) [14, 16], overt OGIB [6, 14, 16], untreated iron deficiency anemia [15, 16], positive SBCE findings [14], and positive BAE findings [16]. Based on these findings, various risk-stratification models have been proposed.

In recent years, two scoring systems have gained attention: the Ohmiya classification system reported by Ohmiya et al. [17] and the RHEMITT score developed by Magalhães et al. [18]. The Ohmiya classification focuses on vascular lesions in OGIB and predicts the rebleeding risk by scoring comorbidities. The RHEMITT score incorporates renal disease, heart disease, endoscopic findings (mainly based on the Saurin classification), bleeding presentation, incomplete SBCE examination, smoking status, and endoscopic therapy. In the scoring system used in this study, P1 and P2 lesions on SBCE were assigned two and three points, respectively, based on the Saurin classification. Our findings demonstrate that P1 lesions, as detected by both SBCE and BAE, were associated with an increased risk of rebleeding. This suggests that revisions to the RHEMITT score may be warranted to more accurately reflect the clinical relevance of P1 lesions identified using multiple endoscopic modalities.

Interestingly, therapeutic intervention, particularly endoscopic hemostasis, is also an independent predictor of rebleeding in patients with SSBB. Patients who underwent a therapeutic intervention had a significantly higher cumulative rebleeding rate than those who did not. This may reflect the complexity of cases in the intervention group, in which the rates of antiplatelet use, vascular lesions, and multiple lesions were higher. These findings suggest that a single endoscopic treatment may not be sufficient to achieve complete hemostasis in such complex cases.

Previous studies have reported that rebleeding after endoscopic hemostasis is common, often due to the presence of multiple lesions and difficulty in identifying the primary bleeding source. In particular, many initial treatments targeted the Yano-Yamamoto classification [19] of type 1a lesions, which were later followed by rebleeding from different lesions. One study reported that among patients initially treated for type 1a lesions, 19% subsequently rebled from type 1b lesions and 25% from type 2 lesions [20]. These findings support the recommendation that repeated endoscopic therapy is necessary to manage vascular lesions in OGIB [20].

In our study, the 3-year cumulative rebleeding rate reached 19.4%, underscoring the importance of long-term follow-up in the management of SSBB, consistent with previous reports [20].

This study had several limitations that should be acknowledged. First, it was a retrospective study conducted at a single center, which may limit the generalizability of the findings. Second, treatment decisions were made at the discretion of attending physicians, introducing a potential selection bias. Third, although both SBCE and BAE were used for lesion detection and treatment, differences in the timing and route of examination may have influenced diagnostic yield and therapeutic outcomes. Finally, because our institutional workflow archives only the finalized consensus report after the double-check process, independent dual readings are not stored. As a result, formal inter-observer agreement measures could not be calculated.

Our findings suggest that modified SC, particularly the presence of P1 lesions, is a significant predictor of rebleeding in patients with SSBB. Therapeutic intervention was also associated with an increased risk of rebleeding, likely reflecting the underlying complexity of the lesions in these cases. These results underscore the importance of risk stratification and long-term follow-up for the management of patients with SSBB.

## Supplementary Information

Below is the link to the electronic supplementary material.

Supplementary file1 (DOCX 15 KB)

## Data Availability

No datasets were generated or analysed during the current study.

## Abbreviations

SSBB: Suspected small bowel bleeding
OGIB: Obscure gastrointestinal bleeding
SBCE: Small bowel capsule endoscopy
BAE: Balloon-assisted endoscopy
ESGE: European society of gastrointestinal endoscopy
modified SC: Modified Saurin classification
EGD: Esophagogastroduodenoscopy
CS: Colonoscopy
CT: Computed tomography
CRP: C-reactive protein
PPI: Proton pump inhibitors
ORs: Odds ratios
IPTW: Inverse probability of treatment weighting
