# Supplementary material for: Long-Term Outcomes and Rebleeding Risk Factors in Suspected Small Bowel Bleeding: A Study Based on the Modified Saurin Classification
Source: Dig Dis Sci. 2026 Jan 4;71(6):2355–64. doi: 10.1007/s10620-025-09640-5 (PMC13357490; doi:10.1007/s10620-025-09640-5)
Supplement: Supplementary file 1 — Supplementary file1 (DOCX 15 KB) [file 10620_2025_9640_MOESM1_ESM.docx]

Supplement table1 Predictors of Re-bleeding Specific to BAE; Multivariate Analysis

| variable | HR (95%CI) | *P* value |
| --- | --- | --- |
| liver cirrhosis | 1.96(0.44-8.71) | 0.37 |
| Antiplatelet drugs use | 2.64 (0.81-8.56) | 0.1 |
| Hemoglobin level (< 10.5 g/dl) | 2.82 (0.83-9.53) | 0.09 |
| modified SC of P1 | 4.14(1.31-13.1) | 0.01 |
| Bleeding type, overt | 1.21 (0.49-3.3) | 0.61 |
| age (>75 years) | 1.51 (0.57-3.94) | 0.4 |
| multiple lesion | 1.68 (0.52-5.38) | 0.38 |
| endoscopic treatment | 1.51 (0.53-4.25) | 0.43 |

BAE, Balloon-assisted endoscopy; SC, Saurin Classification
